# Involvement in Physical Fights among School Attending Adolescents: A Nationally Representative Sample from Kuwait

**DOI:** 10.3390/bs10010029

**Published:** 2020-01-08

**Authors:** Masood Ali Shaikh, Anne P. Abio, Adebola A. Adedimeji, Michael Lowery Wilson

**Affiliations:** 1 Injury Epidemiology and Prevention (IEP) Research Group, Turku Brain Injury Centre, Turku University Hospital and University of Turku, 20014 Turku, Finland; masoodalishaikh@gmail.com (M.A.S.); anne.p.abio@utu.fi (A.P.A.); 2 Department of Epidemiology and Population Health, Albert Einstein College of Medicine, Bronx, NY 10461, USA; adebola.adedimeji@einstein.yu.edu; 3 Injury Epidemiology and Prevention Research Unit, Heidelberg Institute of Global Health (HIGH), University of Heidelberg, 69120 Heidelberg, Germany

**Keywords:** adolescent health, school health, interpersonal violence, epidemiology, bullying, mental health

## Abstract

Background: Interpersonal violence in school settings is an important public health problem worldwide. This study investigated the individual and social correlates for being involved in a physical fight amongst a nationally representative sample of school-attending adolescents in Kuwait. Methods: We carried out bivariate and multivariate analyses to determine the strength and direction of associations with adolescent involvement in problematic fighting behavior within a 12-month recall period. Results: Within a total sample of 3637, *n* = 877 (25.2%) of school-attending adolescents reported being involved in two or more physical fights during the recall period. The multivariate analysis indicated that being male (OR = 2.71; CI = 1.88–3.90), a victim of bullying (OR = 2.77; CI = 2.14–3.58), truancy (OR = 2.52; CI = 1.91–3.32), planning a suicide (OR = 2.04; CI = 1.49–2.78) and food deprivation (OR = 1.91; CI = 1.37–2.65) were associated with an increased risk of involvement in physical fighting. Peer support in the form of having close friends (OR = 0.85; CI = 0.76–0.96) was found to be associated with a reduced involvement in fighting behavior. Conclusion: The results, when taken together, suggest that supportive school environments may represent important settings for violence mitigation and prevention strategies.

## 1. Introduction

Violence among adolescents is a global problem of public health significance. Where violent behavior among adolescents is allowed to persist unabated, it frequently contributes to injury, mental health problems, increased risk behavior, disability, and/or mortality [1]. Violence occurs in various ways and could be in the form of self-harm, which can result in mutilation or suicidal thoughts, interpersonal violence that occurs between one or more persons, and collective violence which occurs among a group of people, for example, political violence [1]. The forms of violence are interrelated with victims potentially turning in perpetrators with time [1]. These behavioral and mental health concerns may also extend into adulthood, where they affect the working and family lives [2]. Youth violence is often linked to bullying, alcohol and substance abuse, as well as gang violence among others. It also contributes to economic losses in areas where it is rampant, increased expenditures in emergency medical care, decreased overall societal well-being, and reduced academic performance in school settings [1]. Some of the most important factors that have been linked with youth violence include: being male, alcohol and/or illicit drug use, bullying victimization, inadequate parental support, truancy, and low socioeconomic status [1,3,4].

Adolescence is a phase when individuals go through development changes and learn to gain independence in various areas [5]. Parental support and connectedness with adolescents have been found to be protective of negative behavior while spending more time with peers can also have a positive or negative influence depending on the character of friends [6]. Parenting styles vary according to different cultures, with an authoritative style being considered acceptable in Arab countries [7]. Supportive friends and favorable school environments also protect against youth violence [4,8,9]. Higher socioeconomic status has also been associated with a reduced risk for interpersonal violence [10]. There are aspects of youth violence that may remain undocumented or unreported—for example, bullying victimization [1]. Bullying victimization has been associated with an increased risk of suicidal behavior and mental health problems among adolescents [11,12]. The victims and perpetrators could also become perpetrators and experience increased emotional stress in their respective social environments [11,12].

Approximately 200,000 cases of homicide occur annually among adolescents and young adults aged 10–29 years, with 83% of the victims being male [1]. Males tend to be overly represented in cases involving youth violence [1,4,8]. Non-fatal violence among youth is equally catastrophic with females getting more involved in non-physical or emotional forms of violence like manipulation or spreading rumors [13]. In one study conducted in Brazil, roughly 20% of injury cases in emergency departments were attributed to interpersonal violence during a one month period in 2006. The population represented in this figure were young people aged 10–19 years [14]. According to several Global School-based Health Surveys (GSHS) conducted between 2003 and 2013, the average prevalence of physical fights among adolescents aged 13–15 was 47% among males and 26% among females with the highest reported in Samoa at 73% and 62% and the least at 21% and 8% in Myanmar, respectively [1].

A higher prevalence of youth violence among school attending adolescents has been reported in the East Mediterranean countries compared to South East Asian countries [15]. Interpersonal violence among adolescents in Kuwait has only recently been examined, with empirical data on the situation in the country being largely inconclusive. For example, Al-Husaini [16] documented that cultural attributes which condone inter-tribal violence as a way of resolving disputes may be a factor in supporting violent behavior among young males. Supportive family structures have also been found to be protective of youth violence in Kuwait [16]. Furthermore, war and family disciplinary practices have also been attributed as factors mainstreaming violence in the region in general [7].

Roughly 40% of male and 34% of female school attending adolescents were involved in physical fights at least once in Kuwait in 2000 [16]. The Iraqi invasion into Kuwait that occurred in 1990 has been blamed for an increase in the youth violence in Kuwait owing to individual experiences; however, an examination of the number of violence cases before and after the invasion showed no difference [16]. Most research on interpersonal violence among adolescents has been derived from high income country settings. Research involving data from the Gulf region remains largely underrepresented. The availability of information is key in the provision of services, especially for adolescents as it is an important phase before transitioning into adulthood. The aim of the present study was to investigate the individual and social correlates for physical fighting among school attending adolescents in the State of Kuwait.

## 2. Methods

### 2.1. Setting

This study was based on data collected in the State of Kuwait, a Western Asian country located at the northern edge of Eastern Arabia, sharing borders with Saudi Arabia and Iraq. It has an estimated 2018 population of about three million, with a 2017 estimated per capita GDP of USD $65,800.

### 2.2. Data Description

The data for this study were derived from the Kuwait contribution to the 2015 Global School-based Health Survey. The methodology for the GSHS was developed collaboratively by the World Health Organization (WHO) and the U.S. Centers for Disease Control and Prevention. All data used in this study are publicly available in anonymized form from the website of the U.S. Centers for Disease Control and Prevention. A two-stage cluster sampling design was used to produce representative data of all students enrolled in grades 8 to 12 in the country. The survey was administered to school attending adolescents in grades 8 to 12, usually attended by those aged 13 to 17 years. It collected self-reported information on indices pertaining to dietary behaviors; mental health; physical activity; protective factors; and violence and unintentional injuries. In the Kuwait 2015 contribution to the GSHS, 3637 students aged 12 years or younger to 18 years or older (weighted percentage: 51.1% female) completed the questionnaire. The overall response rate was 78%, with 97% of schools and 80% of students responding to the survey. The analysis presented in this paper did not exclude any cases; 32 records missing age and 74 records missing sex (16 records were missing both) information were included in the analysis to correctly analyze the data based on the original sampling design.

### 2.3. Measurements

The dependent variable ‘physical fighting’ was derived from the survey question: “*During the past 12 months, how many times were you in a physical fight?*” Response options ranged from “*0 times*”, “*1 time*”, “*2 or 3 times*”, “*4 or 5 times*”, “*6 or 7 times*”, “*8 or 9 times*”, “*10 or 11 times*”, or “*12 or more times*”. For the purpose of our analyses, participants were classified as having participated in a physical fight if they reported being in two or more fights (*n* = 877), within the recall period in order to identify problematic fighting behavior. If none or one fight was reported, participants were classified as not participating in a physical fight (n = 2700); for sixty records, this information was missing.

The classification of fighting behavior into potentially problematic (that which has the possibility to result in short- or long-term seqalae) and potentially non-problematic, admittedly leaves significant room for interpretation. It is, however, understood that, within the context of adolescent development and behavior, disputes arise that may lead to unintended physical altercations [17,18]. Infrequent misunderstandings or “rough play” are not in and of themselves problematic, but are characteristic of rapid bio-psychological, psycho-social and environmental adjustment as adolescents navigate their way into adulthood [19]. The aim with the above classification lies in avoiding pathologizing adolescent development patterns.

We investigated thirteen independent variables to screen for statistically significant associations with the dependent variable; nine at the individual level (age, sex, anxiety, loneliness, bullying victimization, truancy, physical activity, sedentary life style, and suicide planning) and four independent variables at the social level (food insecurity, number of close friends, understanding parents, and helpful peers). All independent variables, apart from age and number of close friends, were dichotomized according to the distribution of the data in order to facilitate analysis (Table 1 and Table 2).

### 2.4. Statistical Analysis

Since there were only 41 students who were either 11 years old or younger and only 44 students who were 12 years of age, they both were combined with the next age category of 13 years for analysis. Univariate analyses characterized the distribution of each selected variable among those who were either never or only once involved in a physical fight versus those who were involved two or more times. This was followed by bivariate analyses utilizing associations among students who were either never or only once involved in a physical fight versus those who reported involvement in two or more physical fights. The categorical and continuous variables were explored with survey versions of a *t*-test and chi-square test. All statistically significant independent variables were included in the multivariable analysis for the dependent variable ‘*physical fight*’ by using the design based multiple logistic regression model.

The logistic regression model included all independent variables found to be statistically significant at *p* < 0.05 in the bivariate analyses. Odds Ratios (OR), including their 95% and 99% confidence intervals, are reported for the strength and direction of associations between involvement in physical fights and the factors studied. Stata version 15 [20] was used for the data analysis.

## 3. Results

Within the recall period, 25.2% (unweighted count: 877) of participants reported being involved in two or more physical fights. The mean age of the entire sample was 15.2 years old (SD: 1.5) and a little less than half (48.9%) were male. Cumulatively, bullying victimization was reported by a quarter (25.2%) of the respondents. Bullying victimization, defined as being bullied one or more days in the past 30 days, was reported by 30.6%—while truancy, defined as having missed classes for 3 or more days in the past 30 days, was reported by 16%. Physical inactivity defined as being physically active for a total of at least 60 min per day for 3 or less number of days in the past seven days was reported by 67%. Not having understanding parents, defined as during the past 30 days parents/guardians either never/rarely/sometimes having understood problems and worries, was reported by 67.5%. Suicide planning, defined as having made a plan about how to attempt suicide in the past 12 months, was reported by 17.4%.

### 3.1. Bivariate Analyses

The bivariate analyses show that, within all the selected independent variables, with the exception of age, and sedentary lifestyle, statistically significant differences existed between students who had been involved in physical fights and those who had not (Table 3). There was no significant difference in the mean age of students who were involved in physical fights when compared with those who were not (15.1 and 15.3 years respectively, *p*-value: 0.10). Proportion of sedentary lifestyle in students who were involved in physical fights when compared with those who were not was the same and not significant (64.5% in both groups, *p*-value: 0.99). In the bivariate analyses, the majority of adolescents reporting involvement in two or more physical fights were males (67.1%).

### 3.2. Multivariable Analyses

An age and sex adjusted analysis (Table 4) was conducted for all variables found to be statistically significant in the bivariate analysis, i.e., 11 out of 13. The analyses revealed statistically significant associations for all selected variables. Males were almost three times more likely to have been involved in physical fights in the past 12 months than females (OR 2.71; 95% CI 1.88, 3.90). Adolescents who experienced bullying were nearly three times more likely to have been involved in physical fights than those who had never experienced bullying (OR 2.77; 95% CI 2.14, 3.58). Truant students were twice as likely to have been involved in physical fights (OR 2.52; 95% CI 1.91, 3.32). Being physically inactive bestowed protection from involvement in physical fights, as physically inactive students were 18% less likely to have been involved in physical fights than those who were physically active (OR 0.82; 95% CI 0.71, 0.95). Not having understanding parents increased involvement in physical fights by 46% (OR 1.46; 95% CI 1.27, 1.67), while suicide planning increased involvement in physical fights by double the amount (OR 2.04; 95% CI 1.49, 2.78). The 99% confidence intervals results show that, with the exception of physical inactivity (99% CI 0.67, 1.01), and helpful peers (99% CI 0.99, 1.87), all other variables were statistically significantly associated with physical fighting status at the *p*-level of <0.01) after adjusting for age and sex.

The final logistic regression model adjusted for all associated covariates that were found to be statistically significant—using a *p*-value of <0.05—in the bivariate analysis was used (Table 5). When compared to those who did not report being involved in a physical fight in the past 12 months, those who had been involved were more likely to be males (OR 2.94; 95% CI 2.09, 4.14), victims of bullying (OR 2.17; 95% CI 1.61, 2.93), less physically active (OR 0.68; 95% CI 0.54, 0.84), truant (OR 1.97; 95% CI 1.44, 2.70), less likely to have understanding parents (OR 1.33; 95% CI 1.12, 1.57), and more likely to have had planned for suicide (OR 1.72; 95% CI 1.41, 2.10). All of these variables were also statistically significant using the 99% CI, i.e., at the *p*-value of <0.01. The goodness-of-fit test revealed that this multivariate logistic model was a good fit for physical fighting in Kuwait among school students (F: 0.65, *p*-value: 0.73). Results based on 99% CIs were consistent with those obtained from the 95% CIs.

## 4. Discussion

Roughly 25% of school attending adolescents reported being in two or more physical fights 12 months preceding the survey. This is similar to estimates reported at 27% in low-middle income countries like India, Indonesia, and Malaysia in studies conducted between 2001 and 2009 [21,22,23], and slightly higher compared to Pakistan at 19% [8]. Studies conducted in the East Mediterranean region have shown the prevalence to range from 46% to 65% among males, 21% to 48% among females, and from 41% to 58% among both sexes [1,15]. Compared to high income countries, the prevalence is lower compared to studies conducted in Israel and Qatar each at 49%, UAE at 43% [15,24,25]; Portugal at 34% and the US at 31% and 36% [15,26,27] though higher compared to other studies conducted in Europe and North America ranging from 9–14% [28,29]. Students involved in physical fights were more likely to be males, involved in bullying victimization, planned suicide, and missed school, while understanding parents and being physically inactive were found to be protective.

Consistent with other studies, being male was significantly associated with physical fighting [4,10,22,29,30]; however, a study conducted in Ghana found no significant difference by gender [9]. Unlike the present study which considered physical fighting to have occurred at least two or more times, the study in Ghana considered gender differences between fights that had occurred once to twice compared to more than three times in the 12 months that preceded the survey [9].

Bullying victimization is also well documented as a risk factor for involvement in violence and physical fights [4,9,22]. Increased bullying among adolescents also led to increased involvement in physical fights in a dose–response relationship in a study in Venezuela [31]. Bullying tends to be fairly common in schools and has ranged from 7% to 72% [31,32,33] and unless interventions are in place to curb the practice, it will contribute to interpersonal violence among youth. Victims are likely to remain silent and may not report it to the relevant authorities for various reasons and extra support may be required for such situations to prevent it from spiralling out of control [34].

Adolescents who missed school were more likely to be involved in physical fight as observed in other studies [4,9,30]. Some students are likely to miss school due to environmental factors for example feeling unsafe at school or on the way to school. The possibility of getting involved in interpersonal violence could lead to a sense of feeling unsafe, which turns into a cycle linked to truancy. The school environment has been reported to be protective of physical fights; however, interpersonal violence in Kuwait may be attributed to tribal beliefs, with students affiliated with tribes more likely to get involved in fights compared to those not affiliated with tribes [15,16].

Interpersonal violence is a risk factor for suicide planning and attempt among adolescents [35,36,37]. This study found an increased risk of suicide planning associated with physical fights. Persons who are violent may be inclined to have suicide tendencies which could remain undetected and ultimately untreated [37]. Concurrent suicide attempts and physical fights among adolescents have also been estimated ranging from 3.89–5.02% in a high income country [37].

Supportive parents were found to be protective of physical fights as observed in other studies in Ghana, Namibia, and Chile [9,28,38] though not in studies from the Caribbean and Malaysia were no association was noted [3,22]. On the other hand, supportive parents were found to have an increased risk among males but protective among females in Qatar [25]. This study measured parental support through how often parents checked the homework of adolescents. Supportive parents have been measured in various ways, for example the studies that used the GSHS surveys have measured it using how often parents understood problems and worries faced by the adolescents [9], although it was not clear how the variable was measured in the other two studies [28,38]. Studies conducted in Arab countries have found that bonding between adolescents and parents is not the same as in Western countries [7]. The culture in Arab countries is more collective where interdependence within a family emphasized, an authoritative parenting style is used and not associated with negative outcomes [7].

Physical inactivity was found to be protective of physical fights, which is surprising as physically active adolescents were more likely not to fight in a study conducted in high income countries [29]. A study conducted in Egypt found no association for physical activity [4]. The recommended at least 30 min of physical activity on at least moderate intensity on most, preferably all, days per week [39]. It is estimated that approximately 80% of adolescents aged 13–15 years undertake less than 60 min of physical activity daily [40,41]. This study considered three days or less per week to constitute physical inactivity, thus adolescents with the minimum recommendation physical activity may have been classified as physically inactive.

No statistically significant association was found with age as noted in other studies in Ghana and Namibia [9,38], while studies conducted in both low- and middle-income settings noted an inverse relationship between physical fights and age [4,10,23,26,29,30].

This study found no association between socioeconomic status and physical fights. Socioeconomic status has been associated with physical fighting [10,33,42], though no association was found in a study in Ghana [9], in the Caribbean [3], and among males in Canada [27]. The studies in the Caribbean, Ghana, and the current study used hunger as a measure of socioeconomic status while other studies have used self-reported affluence, family affluent scale, or average household income as a unit of measurement. Additionally, no association between physical fights was found between anxiety, sedentary lifestyle, loneliness, and close friends as noted in other studies [3,4]. In a study in India, adolescents who reported feeling lonely most of the time were more likely to fight [43].

### Strengths and Limitations

The research has contributed to knowledge about interpersonal violence in Kuwait using the 2015 GSHS survey. To our knowledge, this is the first study that examined the associations between physical fighting among adolescents and other known risk factors in Kuwait. It is anticipated that the information could be used to inform policy and recommendations on necessary adolescent health with regards to interpersonal violence, especially within the school environment.

The study has some limitations that need to be taken into consideration. The survey was conducted among adolescents in school, and may not adequately reflect factors associated with physical fighting for those who do not attend school. The study was cross-sectional and causal relationships between variables could not be estimated. Additionally, the study was not able to estimate the cause of physical fights among students or who the students fought with.

## 5. Conclusions

Considering that being male and being bullied were strongly associated with physical fighting behavior in Kuwait, interventions which target males and bullying may be necessary to address interpersonal violence among adolescents. Reduction in the physical fighting behavior may also lead to a reduction school absences. Ultimately, the creation of an environment where all students feel safe may enable those who attend school to adequately learn and concentrate in the school environment. The US Centers for Disease Control advocates for a dynamic multi-faceted approach to violence prevention by addressing factors at all levels of the social ecology—the individual, relational, community, and societal levels. Specifically, the following Table 6) is instructive:

## Figures and Tables

**Table 1:** Independent variable derivation from the GSHS survey data in Kuwait 2015.

Survey Question	Coding	Variable
**Individual level variables**		
How old are you?	13–18 years (continuous)	Age
During the past 30 days, on how many days were you bullied? **Responses**: 0 days, 1 or 2 days, 3 to 5 days, 6 to 9 days, 10 to 19 days, 20 to 29 days, all 30 days	0 days (0); 1 or more days (1)	Bullying victimization
During the past 7 days, on how many days were you physically active for a total of at least 60 min per day? **Responses**: 0 days, 1 day, 2 days, 3 days, 4 days, 5 days, 6 days, 7 days	4 to 7 days (0); 3 days or less (1)	Physical inactivity
How much time do you spend during a typical or usual day sitting and watching television, playing computer games, talking with friends, or doing other sitting activities such as Dewaneya; using mobile or tablet devices to play games or chat using social media like Instagram, Snapchat, or Twitter; playing cards; or going to the cinema? **Responses**: less than 1 h per day, 1 to 2 h per day, 3 to 4 h per day, 5 to 6 h per day, 7 to 8 h per day, more than 8 h per day	2 h or less per day (0); 3 to 8 or more hours per day (1)	Sedentary lifestyle
During the past 12 months, how often have you been so worried about something that you could not sleep at night? **Responses**: never, rarely, sometimes, most of the time, always	(Never, rarely, sometimes = 0); (Most of the time, always = 1)	Anxiety
During the past 12 months, how often have you felt lonely? **Responses**: never, rarely, sometimes, most of the time, always	(Never, rarely, sometimes = 0); (Most of the time, always = 1)	Loneliness
During the past 30 days, on how many days did you miss classes or school without permission? **Responses**: 0 days, 1 or 2 days, 3 to 5 days, 6 to 9 days, 10 or more days	Zero to 2 days (0); 3 to 10 or more days (1)	Truancy
During the past 12 months, did you make a plan about how you would attempt suicide? **Responses**: yes, no	No (0); Yes (1)	Suicide Planning

**Table 2:** Independent variable derivation from GSHS survey data Kuwait 2015.

Survey Question	Coding	Variable
**Social-Level Variable**		
During the past 30 days, how often did you go hungry because there was not enough food in your home? **Responses**: never, rarely, sometimes, most of the time, always	(Never, rarely, sometimes = 0); (Most of the time, always = 1)	Food insecurity
During the past 30 days, how often did your parents or guardians understand your problems and worries? **Responses**: never, rarely, sometimes, most of the time, always	(Most of the time, always = 0); (Never, rarely, sometimes = 1)	Understanding parents
During the past 30 days, how often were most of the students in your school kind and helpful? Responses: never, rarely, sometimes, most of the time, always	(Most of the time, always = 0); (Never, rarely, sometimes = 1)	Helpful peers
How many close friends do you have? **Responses**: 0, 1, 2, 3 or more	0 = (0); 1 = (1); 2 = (2); 3 or more (3)	Close friends (continuous)

**Table 3:** Distribution of selected factors according to categories of physical fighting among school-attending adolescents in Kuwait, GSHS 2015.

Variable	Not Involved in Physical Fights (*n* = 2700)	Involved in Physical Fights (*n* = 877)	*p*-Value
Age (SD)	15.3 (1.5)	15.1 (1.5)	0.104
Sex (male)	43.0	67.1	<0.001
Bullying victimization	24.6	48.3	<0.001
Physical inactivity	68.7	61.5	0.002
Sedentary lifestyle	64.5	64.5	0.987
Anxiety	20.0	24.1	0.013
Loneliness	18.3	22.8	0.009
Truancy	12.9	24.9	<0.001
Suicide planning	14.9	25.1	<0.001
Food deprivation	6.9	10.2	0.016
Understanding parents	65.6	72.9	<0.001
Helpful peers	55.4	63.1	0.005
Close friends (SD)	2.4 (0.93)	2.3 (0.99)	0.030

All variables are expressed as proportions (in %) with the exception of ‘age’ and ‘close friends’ which are expressed as mean and standard deviation.

**Table 4:** Multivariable analysis of physical fighting among school-attending adolescents in Kuwait, GSHS 2015.

Variable	OR	95% CI (99% CI)	*p*-Value
Sex (male)	2.71	1.88, 3.90 (1.64, 4.48)	<0.001
Bullying victimization	2.77	2.14, 3.58 (1.94, 3.95)	<0.001
Physical inactivity	0.82	0.71, 0.95 (0.67, 1.01)	0.013
Anxiety	1.57	1.29, 1.91 (1.19, 2.06)	<0.001
Loneliness	1.56	1.26, 1.93 (1.16, 2.09)	<0.001
Truancy	2.52	1.91, 3.32 (1.72, 3.69)	<0.001
Suicide planning	2.04	1.49, 2.78 (1.32, 3.13)	<0.001
Food deprivation	1.91	1.37, 2.65 (1.21, 3.01)	0.001
Understanding parents	1.46	1.27, 1.67 (1.20, 1.76)	<0.001
Helpful peers	1.37	1.09, 1.71 (0.99, 1.87)	0.010
Close friends	0.85	0.76, 0.96 (0.72, 0.99)	0.010

OR: Odds Ratio 95% CI and 99% CI: 95% and 99% Confidence Intervals. All estimates are adjusted for age and sex; while sex was adjusted for age.

**Table 5:** Outcomes of multivariable analysis of variables associated with physical fighting attempts among school-attending adolescents in Kuwait, GSHS 2015.

Variable	OR	95% CI (99% CI)	*p*-Value
Sex (male)	2.71	1.88, 3.90 (1.64, 4.48)	<0.001
Bullying victimization	2.77	2.14, 3.58 (1.94, 3.95)	<0.001
Physical inactivity	0.82	0.71, 0.95 (0.67, 1.01)	0.013
Anxiety	1.57	1.29, 1.91 (1.19, 2.06)	<0.001
Loneliness	1.56	1.26, 1.93 (1.16, 2.09)	<0.001
Truancy	2.52	1.91, 3.32 (1.72, 3.69)	<0.001
Suicide planning	2.04	1.49, 2.78 (1.32, 3.13)	<0.001
Food deprivation	1.91	1.37, 2.65 (1.21, 3.01)	0.001
Understanding parents	1.46	1.27, 1.67 (1.20, 1.76)	<0.001
Helpful peers	1.37	1.09, 1.71 (0.99, 1.87)	0.010
Close friends	0.85	0.76, 0.96 (0.72, 0.99)	0.010

OR: Odds Ratio 95% CI and 99% CI: 95% and 99% Confidence Intervals. All estimates are adjusted for age and sex; while sex was adjusted for age.

**Table 6:** U.S. Centers for Disease Control violence prevention recommendations.

Item 1	Item 2
Promote family environments that support healthy development	Early childhood home visitation and the parenting skill and family relationship programs
Provide quality education early in life	Preschool enrichment with family engagement
Strengthen youth’s skills	Universal school-based programs
Connect youth to caring adults and activities	Mentoring programs and after-school programs
Create protective community environments	Modify the physical and social environment, Reduce exposure to community-level risks, Street outreach and community norm change
Intervene to lessen harms and prevent future risk	Treatment to lessen the harms of violence exposures, Treatment to prevent problem behavior and further involvement in violence, Hospital-community partnerships

Excerpted from https://www.cdc.gov/violenceprevention/youthviolence/prevention.html.
